# Study protocol: assessment of the usefulness and practicability of a psychoeducational intervention to prevent the negative psychological impact of the COVID-19 pandemic on primary care health workers

**DOI:** 10.1186/s12875-023-02187-2

**Published:** 2023-11-04

**Authors:** Enric Aragonès, Sara Rodoreda, Meritxell Guitart, Eva Garcia, Anna Berenguera, Francisco Martin, Concepció Rambla, Guillem Aragonès, Antoni Calvo, Ariadna Mas, Josep Basora

**Affiliations:** 1 Research Unit. Primary Care Area Camp de Tarragona, Institut Català de la Salut, Tarragona, Spain; 2 Institut Universitari d’Investigació en Atenció Primària Jordi Gol (IDIAPJGol), Barcelona, Spain; 3 Centre d’Atenció Primària de Constantí, Carrer dels Horts, 6, Constantí, Tarragona 43120 Spain; 4 Primary Care Division, Institut Català de la Salut, Barcelona, Spain; 5 Primary Care Emotional and Community Wellbeing Program, Department of Health, Government of Catalonia, Barcelona, Spain; 6 Primary Care Centre Sant Vicenç de Castellet, Institut Català de la Salut, Barcelona, Spain; 7 Occupational Risk Prevention Unit, Institut Català de la Salut, Barcelona, Spain; 8 School of Medicine and Health Sciences, Universitat Rovira i Virgili, Reus, Spain; 9 Galatea Foundation, Barcelona, Spain; 10 Department of Health, General Directorate of Health Planning and Research, Government of Catalonia, Barcelona, Spain

**Keywords:** Mental health, Health workers, Primary healthcare, COVID-19 pandemic, Burnout, Psychological support

## Abstract

**Background:**

The COVID-19 pandemic has constituted an extraordinarily stressful situation for healthcare professionals and has led to psychological distress and an increase in various mental disorders. In the post-pandemic context, it is necessary to provide professionals with strategies and skills to manage this stressful situation and prevent or minimize its negative impact.

**Methods:**

*Aims*: To assess the feasibility and clinical effects of a group psychoeducational program focused on preventing the adverse psychological and emotional effects of the pandemic on primary care workers, and to explore the experience and perceptions of participants with regard to the program from a qualitative perspective. *Design*: A single-arm, before-and-after study conducted in primary care. *Setting*: The 332 primary care centers of the Catalan Institute of Health (Catalonia, Spain) *Participants*: The target population of the intervention is primary care workers, including clinical profiles (e.g., nurses and doctors), and non-clinical profiles (e.g., administrative staff). The implementation strategy will also involve community psychologists, who will lead the psychoeducational groups, and the health organization promoting the implementation. *Intervention*: A group psychoeducational program targeting primary care workers to promote emotional well-being and the ability to cope with stressful situations. Community psychologists will deliver it in the primary care centers they are linked to. *Measures*: Mixed-methods evaluation, combining quantitative and qualitative research. A prospective assessment of the main outcomes (professional quality of life, psychological state, and resilience) will be performed using online questionnaires before and immediately after the intervention, and at 3 and 6 months. A qualitative study will be conducted, comprising focus groups and individual in-depth interviews with the participants in the intervention and the psychologists who provide it. *Ethics*: The Research Ethics Committee of the Jordi Gol Primary Care Research Institute (IDIAP) has approved the protocol (22/086-PCV).

**Discussion:**

This project proposes an intervention to promote mental health and psychological well-being in primary care workers by learning skills and integrating them into personal and professional life. The expected results will allow us to determine the usefulness and effectiveness of this psychoeducational intervention under the conditions of real clinical practice, provide data to model and perfect it, and promote its dissemination.

**Trial registration:**

ClinicalTrials.gov Identifier: NCT05720429; registered on 09/02/2023.

## Background

The COVID-19 pandemic has forced healthcare workers to face very intense stressful situations at the professional, personal and family level. In the work setting, professionals have endured extended workdays, work overload, the permanent need to concentrate and remain alert, and strict security measures, as well as the occasional shortage of protective equipment. Many workers have had to perform tasks for which they were not prepared, which, together with the sustained risk of infection due to contact with patients, has resulted in very high emotional stress [1, 2].

To adapt to the health crisis, significant organizational changes were implemented in primary care settings within the Catalan health system, including restrictions on in-person visits and the provision of health care via telephone, the creation of special circuits for potential COVID-19 patients, as well as home care for these patients, and the adoption of new roles, such as for screening and case and contact tracing, the out-of-hospital management of most COVID-19 patients, and health care in nursing facilities [3–5].

This long-lasting situation of stress has put both the physical and mental health of professionals at risk. Symptoms of anxiety, depression and stress have been observed in healthcare professionals, as well as an increase in the incidence of psychological disorders [6, 7]. A study conducted in 2020 on the psychological state of primary care workers found that 44% screened positive for a mental disorder and that some professional profiles, including administrative workers and nurses, were especially at risk for adverse mental health [8].

Burnout is a syndrome resulting from workplace stress and is characterized by the following symptoms: feelings of exhaustion, increased mental detachment or feelings of negativism or cynicism related to one’s job and patients, and reduced satisfaction with one’s job. Luceño-Moreno et al. [9] reported high levels of burnout in a sample of healthcare workers in Spain in the context of the pandemic, especially in the dimension of emotional exhaustion, which affected 41% of the sample. In a prospective study on a cohort of primary care physicians in Catalonia, Seda-Gombau et al. [10] observed an extraordinary increase in the levels of burnout during the pandemic compared to previous figures. Emotional distress and burnout among healthcare workers are highly pertinent concerns. These conditions are significant not only due to the emotional hardship experienced by the affected individuals but also because they can potentially lead to a decline in the quality and effectiveness of their work, thereby affecting patient safety [11, 12].

While the pandemic is now ostensibly under control, the situation has exposed many deficiencies in the health system, which is still under intense pressure especially at the primary care level [13]. The psychological vulnerability of healthcare workers has also been underscored, and moreover, exhaustion and the psychological consequences of this experience may appear at any time and be long-lasting [14].

In this context, it is appropriate and necessary to develop interventions to promote the mental health of this primary care collective, not only to ensure the well-being of primary care workers, but to also guarantee the quality of their work and the safety of patients [11, 15].

## Methods/design

### Aims

This study aims to explore the implementation of a group psychological intervention to promote mental health and prevent and manage psychological distress in primary care workers with regard to the COVID-19 pandemic.

This general objective is broken down into the following specific objectives: (1) measure the clinical effects of the intervention in terms of resilience, psychological symptoms, burnout, and professional quality of life; (2) identify predictors of the clinical effects of the intervention; (3) explore, from a qualitative perspective, the experience and perceptions of the participants (including both the healthcare workers who receive the intervention and the psychologists who deliver it) regarding the feasibility, usefulness, effectiveness and integration of the skills learned into their work and personal lives, and identify facilitators, barriers and proposals to improve the program and its implementation; and lastly, (4) shape the psychoeducational program and the implementation strategy based on the results obtained from the evaluation of it.

### Design

This is a single-arm study of the implementation of a healthcare intervention with a pre and post-intervention design, using a mixed methods approach that combines both qualitative and quantitative data analysis [16]. The design involves the implementation and evaluation of the intervention in real clinical practice.

### Settings and participants

The scope of this project is the 332 primary care centers of the Catalan Institute of Health, which cover approximately 75% of the inhabitants of Catalonia (Spain) [17]. The target population is the staff of these primary care centers, comprising all professional profiles of the primary care teams: primary care nurses, family doctors, pediatricians, dentists, physiotherapists, nutritionists, social workers and administrative staff [18]. In Catalonia’s primary care system, administrative staff in the primary care centers, although they are non-clinical professionals are recognized as integral members of the Primary Care Team. They are responsible for tasks such as patient reception, guidance, and assistance. Furthermore, a study focusing on the emotional toll of the pandemic on primary care personnel identified administrative staff as a vulnerable group with a heightened risk of experiencing adverse mental health effects [8].

Having a severe mental disorder or being in the process of litigation for work disability due to a psychological disorder are exclusion criteria for the evaluation process.

### Intervention

The intervention consists of a group psychoeducational program aimed at primary care professionals developed at the initiative of the Directorate of Primary Care of the Catalan Institute of Health as part of the Program for Emotional Well-being and Community Health in Primary Care (Department of Health, Generalitat de Catalunya) [19]. It was designed in collaboration with working groups formed by psychologists with expertise in the various areas included in the program. Based on a selection of psychological and psychoeducational interventions backed by scientific evidence, a toolbox was created, or rather, a set of strategies that primary care professionals can integrate into their personal and professional lives to promote psychological well-being and prevent adverse mental health. The program also aims to create safe spaces for emotional venting to reduce the emotional burden and to improve group cohesiveness and peer support.

The sessions will be delivered by the community psychologists linked to the primary care centers as part of the aforementioned Emotional Well-being Program. The psychoeducational program will be offered at all the primary care centers of the Catalan Institute of Health and will be open for participation to the personnel of these centers. It consists of 11 in-person weekly or biweekly sessions lasting 45 to 60 min, which will be held at the primary care centers during the professionals’ work hours. The content of the sessions is structured as follows: a brief introduction to the theory behind the concept to be discussed, a practical part where activities related to the topic of the session are conducted and, lastly, a brief guided meditation. Table 1 presents the session topics and contents. Although the structure and specific contents of each session are defined in the protocol, it is expected that the program may be modified or adapted based on local preferences, conditions, and needs (e.g., the number and/or order of sessions, frequency or structure of the sessions, or the addition of new topics). In a subsequent phase, it is anticipated that these group sessions will be incorporated into the regular routine of primary care centers, providing professionals with the opportunity to focus on their emotional well-being through a proactive approach to preventing and promoting mental health, which will be sustained beyond the scope of this project.

**Table 1:** Index of sessions of the psychoeducational program

Title	General content
Emotional management	Identification and regulation of emotions.
Thought management	Inner dialogue, irrational beliefs, rational thoughts vs. irrational thoughts.
Stress management	What is stress, why does it occur and how to deal with it? Stress and health.
Communication skills	Assertiveness and communication styles, empathy, active listening.
Self-care	Definition and importance, dimensions of self-care.
Individual and group self-esteem	Definition, importance of self-esteem, self-esteem and self-concept, characteristics of low self-esteem.
Anxiety/coping with panic Mindfulness	Concepts of anxiety and stress. Introduction to mindfulness.
Activate motivation	Theoretical basis of motivation. Self-motivation strategies.
Problem-solving	Definition of concept. Ways to deal with conflicts. Problem-solving techniques.
Positive psychology and emotional intelligence	Positive psychology. Positive emotions and resilience. Effectiveness and positive attitudes at work. Engagement. The process of “flowing”.
Emotional expression through art	Health benefits of art.
Each session adheres to a defined structure, which includes the following components: (1) Initial content exposition led by the facilitator; (2) Interactive activities designed to reinforce the session’s content, (3) Guided relaxation exercises; and (4) Reminders of available resources for participants.	

### Intervention: implementation strategy

We have developed an implementation strategy based on the PARIHS model (Promoting Action on Research Implementation in Health Services) [20]. According to this theoretical and operational framework, successful implementation is dependent on three factors: evidence that supports the proposed program, the context in which the new program is to be applied, and the facilitation factors that drive and maintain it.

The PARIHS framework recognizes evidence that supports implementation in a broad sense, including both explicit sources of evidence (i.e., published research) and implicit evidence from other sources such as the experience and knowledge of the psychologists conducting the psychoeducational program. The opinions and preferences of the healthcare workers receiving the intervention are also included as sources of evidence. There is evidence on the effectiveness of interventions to promote resilience and psychological well-being in health workers, which will be reviewed and evaluated [21, 22]. Implicit knowledge will be gathered through qualitative techniques, such as individual and group interviews with leading professionals in the fields of primary care, mental health, and occupational health.

The context, includes the characteristics of the healthcare organization (i.e., receptivity, culture of innovation, leadership, available resources) that promote implementation and the barriers that the implementation team should investigate, identify, and manage. One aspect of our context that contributes to the likelihood of successful implementation of the program is the institutional commitment of the Primary Care Division of the Catalan Institute of Health to it as a priority project.

Facilitation refers to the support provided to achieve the effective implementation of the psychoeducational program. The management positions of the health institution involved in the deployment of the program, as well as the local and regional managers of the network of community psychologists will be appointed internal facilitators (i.e., from within the health organization). Strategies will be developed to foster a sense of belonging, involvement and commitment to the project among this network of community psychologists. External facilitation will be conducted by the core implementation team (linked to the research team promoting this project). They will carry out the tasks of training, technical support, advice, evaluation, feedback, adaptation of the intervention to the local context, accreditation, and inter-institutional coordination, among others.

### Measurements

#### Procedure

We will evaluate a set of quantitative indicators covering various aspects of the care process:

- Number of editions of the program held during the study period.
- Number of primary care centers where at least one edition of the program was held over the total number of primary care centers.
- Number of editions during which at least 6 of the 11 standard program sessions were held.
- Number of healthcare professionals who participated in the program over the target population, both in total and by professional profile.
- Percentage of participants who attended at least 6 sessions, in total and by professional profile.

The outcomes will be prospectively assessed individually, with designated assessment points established at the baseline, i.e., before starting the psychoeducational intervention, immediately following the intervention, at 3 months and 6 months after the intervention (Table 2). Data will be collected through standardized online questionnaires that participants will complete independently.

**Table 2:** Study variables

Assessment area	Instrument	Time of assessment ^a^			
		Tpre	T0	T3	T6
***Healthcare workers participating in the intervention***					
Age, sex, marital status, family characteristics	Sociodemographic data form	x			
Profession, employment status, seniority in the workplace, health center where they work	Workplace characteristics form	x			
General health status	SF-12 questionnaire (item 1)	x			
Previous and/or current mental disorders, psychopharmacologic or psychological treatment	Mental health form	x			
Professional quality of life: compassion satisfaction, perceived support, burnout, secondary traumatic stress, and moral distress	ProQOL	x	x	x	x
Psychological state: depression, anxiety, and stress	Depression, Anxiety, and Stress scales (DASS-21)	x	x	x	x
Resilience	Connor-Davidson Resilience scale (CD-RISC 10)	x	x	x	x
Level of attendance to the program sessions	Self-reported form		x		
Participants’ satisfaction with and suggestions for improvement of the program	Self-reported form		x		
Participants’ perceptions of usefulness and feasibility of learned skills in their personal and professional life	Self-reported form		x	x	x
***Community psychologists***					
Actual adherence to the standard program and achievement of objectives	Quality checklist		x		
***Qualitative methods***					
Facilitators, barriers and proposals for improvement	Focus groups with community psychologists		x		
	Focus groups and in-depth interviews with participants		x		

^a^ The assessment points are established at baseline, i.e., before the intervention (Tpre), immediately after the intervention (T0), at 3 months (T3) and at 6 months (T6)

### Outcomes: baseline measurements and prospective follow-up

- **Professional quality of life in the psychological area**: This will be measured with the ProQOL [23, 24], a questionnaire with 30 items that explore the feelings and perceptions of health professionals regarding their work and which are answered on a Likert scale for frequency, from 1 (“never”) to 5 points (“always”). The following dimensions are evaluated: compassion satisfaction, which measures the satisfaction derived from being able to do work well and care for patients well; burnout, associated with feelings of hopelessness, exhaustion and difficulty meeting the demands of the job; and secondary traumatic stress, which is related to secondary exposure to stressful events in the workplace. The ProQOL shows psychometric goodness in its Spanish version, and Cronbach’s alphas in Spanish healthcare workers were reported to be 0.87 for compassion satisfaction, 0.70 for burnout, and 0.84 for secondary traumatic stress. [24, 25].
- **Psychological state**: This will be measured with the Depression, Anxiety and Stress Scales (DASS-21) [26, 27]. The DASS-21 contains three scales that assess the presence of symptoms and indicators of depression, anxiety and stress. Each scale contains 7 items that are rated on a Likert scale from 0 points (“did not apply to me at all”) to 3 points (“applied to me very much or most of the time”). Psychometric features of the Spanish version of DASS-21 are comparable to those of the original English version. Data on internal consistency are good with high subscale coefficient alphas (0.93 for depression, 0.86 for anxiety, and 0.91 for stress) [27].
- **Resilience**: Resilience is a process in which an individual develops adaptive skills in the face of adverse situations. This construct will be measured with the Connor-Davidson Resilience Scale (CD-RISC 10) [28, 29], which has 10 items with statements about behaviors and attitudes that are considered to denote resilience in the respondent. They are scored on a Likert scale from 0 points (“Not true at all.”) to 4 points (“True nearly all the time.”), such that higher scores indicate greater resilience. The Spanish version of the CD-RISC 10 has shown to provide valid and reliable data with a good internal consistency when used in a sample of workers (Cronbach’s alpha = 0.87) [29].

### Secondary outcomes: prospective follow-up using self-reported forms

- Compliance with program sessions (i.e., the number of sessions attended by the participant.).
- Satisfaction with and personal evaluations of the program, answered by means of an ad hoc questionnaire with five-point Likert responses ranging from “completely disagree” to “completely agree”, including items on participants’ perceptions of the suitability of the objectives, content, and methodology of the program, and on the quality of the psychologist who delivers it.
- Usefulness and practicability of the skills learned in their personal and professional lives, measured by means of an ad hoc questionnaire with five-point Likert responses, ranging from “completely disagree” to “completely agree”.

**Explanatory variables: baseline measurements**.

- Sociodemographic and work characteristics: age, sex, marital status, profession, employment status, seniority in the workplace, health center where employed.
- Self-assessment of general state of health, using the first item on the SF-12 survey [30], which classifies general health into five categories: excellent, very good, good, fair and poor.
- Past and current mental disorders, and current psychopharmacological and/or psychotherapeutic treatment.
- Contact with COVID-19 patients due to work (never, occasionally, frequently, continuously).
- History of COVID-19 infection, severity and time elapsed since infection.
- History of persistent COVID-19 symptoms, defined as symptoms lasting more than three months.

### Analysis plan

An initial analysis will be performed using standard statistical methods to describe the characteristics of the sample, specifically the prevalence and characteristics of the various emotional and psychological symptoms studied, as well as the factors associated with them.

Regarding the prospective follow-up of the cohort, an analysis will be conducted of the evolution of the variables related to participation in the psychoeducational activity. These analyses will include the evolution of the various emotional symptoms studied and the identification of predictive factors. Bivariate and multivariate analyses will be performed using logistic or linear regression models depending on the evaluated outcome to identify predictive factors of the evolution of the psychological variables. The level of significance will be set at p < 0.05 and ORs or mean differences will be reported as appropriate, as well as corresponding p values and 95% confidence intervals.

### Qualitative assessment

A theoretical study with a phenomenological approach will be conducted to understand participants’ perceptions and evaluations of their experience of the psychoeducational intervention. Participants in the qualitative study will be recruited from among (a) the psychologists conducting the new intervention, and from among (b) the professionals participating in the psychoeducational activity. Each profile will be analyzed independently.

The sampling will be theoretical, based on individuals who have expressed their willingness to participate in the qualitative study. They will be selected based on certain criteria to achieve maximum variety of discourse: (a) psychologists: geographic scope, size and rural or urban status of the primary care center, and degree of adherence to the standard psychoeducational program, and (b) health workers: sex, profession, seniority, geographic scope, level of impact to emotional state, and degree of adherence to the program activities.

The data will be collected through group and individual interviews conducted online. Group interviews will last 90 min, be moderated by a qualified researcher and have an observer. Individual interviews will be conducted with the participants who present the greatest emotional impact. Both individual and group interviews will preferably be conducted online using Microsoft Teams.

The topic script will include concepts such as the usefulness, feasibility and possibility of integrating the tools and strategies learned in the psychoeducational intervention into one’s personal and professional life. Obstacles and difficulties will be explored as well as any suggestions and proposals for improvement that might help us mold and perfect the intervention. The content of the sessions will be video and audio recorded and will later be transcribed literally and in full for analysis.

We will use thematic framework analysis to classify and organize the data according to key topics, concepts and predefined constructs. These will be analyzed using qualitative methods adapted from normalization process theory to identify barriers and facilitators in the different areas, delving into “hot spots” such as controversial issues and uncertainties.

A minimum of two group interviews with health workers and two with psychologists will be held, although as many as needed will be conducted until data saturation is reached.

### Trial status and schedule

This study was registered with ClinicalTrials.gov with the identifier: NCT05720429 on February 9, 2023. Implementation of the psychoeducational program will run from September 2022 to December 2023; the primary completion date (i.e., date on which the last participant in the clinical study will be examined) will be June 2024; the qualitative analysis will be performed from April 2023 to May 2024; the analysis and publication of the results will take place from June 2024 to December 2024; the modeling and improvement of the psychoeducational program based on the results of the evaluations will occur between June 2024 and December 2024.

In accordance with the project schedule, the research team has already designed the psychoeducational program, edited and published the intervention manual and didactic materials. The authors are doing dissemination and information about the program, and a training course for psychologists to facilitate homogeneous deployment is currently being finalized. Some centers have started their first psychoeducational groups.

### Ethics

The study was designed in accordance with the Guide to Good Practice in Health Science Research [31] and the principles of the Declaration of Helsinki of the World Medical Association, modified in 2013 and the applicable regulations. The protocol was approved by the Jordi Gol IDIAP Ethics Committee (Barcelona, 27/05/2022; code 22/086-PCV).

This study explores the usefulness of an intervention performed in real practice, with voluntary recruitment and participation in a psychoeducational activity. Participants will be informed of the objectives and general aspects of this study and informed consent to participate will be obtained from all the participants. The informed consent will specifically include voluntariness, data security and confidentiality and their exclusive use for research purposes. It will also include non-maleficence for participants and the possibility of participating in the psychoeducational intervention without taking part in the evaluation. If, while participating in the program or study evaluation, significant situations of mental distress arise, circuits have been established to appropriately assess and approach them in the corresponding health facility.

## Discussion

The pandemic has had an intense impact on the psychological state of healthcare workers and, specifically, primary care professionals [6, 8]. Mental distress in healthcare workers can have significant implications in their personal and family well-being, and the effectiveness and quality of their work [32]. Since this project was conceived, the COVID-19 pandemic has evolved [33] and the current epidemiological status of the pandemic and its repercussions on healthcare professionals and the healthcare system are currently qualitatively and quantitatively different. However, the pandemic has revealed deficiencies, shortcomings and tensions in healthcare systems [34] and, above all, the need to attend to the psychological well-being of healthcare workers is now more evident than ever [35]. In this context, the research project described in this article is particularly relevant and timely.

The project is an example of translational research and attempts to bridge the gap between scientific knowledge and clinical practice. The program to be evaluated is a compendium of tested psychological interventions that are well established in the practice of psychology and supported by scientific evidence [36–38]. However, they have not been tested and evaluated under the conditions in which they will be implemented in this project: to the entire primary care system of Catalonia under the conditions of real clinical practice. The implementation strategy was developed following the PARIHS framework [20], which constitutes both a solid theoretical framework and an operational guide for structured implementation procedures and their evaluation.

One of the strengths of this initiative is its wide scope, since it targets more than 300 primary care centers of the Catalan Institute of Health. Moreover, the commitment and active involvement of this institution in prioritizing and deploying this intervention increases the chances of it being implemented successfully.

The project also has limitations to consider. Firstly, the quasi-experimental design without a control group may represent a limitation regarding the evaluation of the procedure in terms of clinical effectiveness. However, the proposed methodology is completely appropriate for a implementation study [39, 40], where the objective is not so much to evaluate the effectiveness of the intervention, but rather focuses on concepts such as practicability in the context of real clinical practice, its feasibility, its acceptability and integration into the daily operations of primary care centers and their professionals, and the possibility of adapting the procedure to different realities and healthcare situations. Secondly, voluntary participation may lead to a self-selection bias in the participants [41]. Thirdly, our psychoeducational intervention is primarily designed to improve emotional well-being and does not explicitly address aspects of job performance. Nevertheless, studies have consistently demonstrated that the emotional well-being of workers can exert a significant influence on their performance and work outcomes [11, 12], which in turn can have repercussions on patient safety. We acknowledge that our project design has a limitation in this area—it does not encompass the quantitative measurement of outcomes directly linked to work performance or patient safety. However, we will have the opportunity to explore these concepts in the qualitative assessments. Fourthly, a limitation inherent to the design of the proposed intervention is that the personal factors of resilience are addressed, but there is no intervention, at least directly, on the contextual factors—workload and working conditions, organization, team dynamics, etc.—that can be decisive in the well-being or mental distress of professionals [42, 43]. In light of this risk, procedures have been contemplated to collect the concerns of professionals that go beyond the objectives and scope of the program and transfer them to the competent authorities. Subsequently, the participants will be able to help model the intervention based on their experience, preferences and conditions.

The results we anticipate, derived from the synthesis of both quantitative and qualitative data, will provide us with a comprehensive understanding of the impact of the psychoeducational intervention. Our evaluation will encompass not only its effectiveness but also the perceived feasibility, utility, and possibilities for improvement by both the psychologists facilitating the groups, as well as the healthcare workers involved. These findings will be interpreted and used to redesign and improve both the intervention and the implementation strategies, fostering a continuous improvement process.

We predict that implementing this intervention will prompt substantial changes in how primary care workers deal with stress and complex situations, which are not uncommon in their work. We also expect the implementation and dissemination of the program to have a global impact, which could be very relevant considering the high prevalence of emotional distress among healthcare workers and the repercussions of this reality on the quality and effectiveness of their work, the safety of patients and the functioning of the healthcare system in general.

## Data Availability

The data that will be generated by this study will be made available upon reasonable request to the principal investigator (EA).

## List of abbreviations

CD-RISC10: Connor-Davidson Resilience Scale 10 items
COVID-19: Coronavirus Disease of 2019
DASS-21: Depression, Anxiety and Stress Scales, 21 items
ICS: Catalan Health Institute (in Catalan:Institut Català de la Salut)
IDIAP: Institute for Primary Health Care Research (in Catalan:Institut d’Investigació en Atenció Primària)
PARIHS: Promoting Action on Research Implementation in Health Services
PERIS: Strategic Plan for Research and Innovation in Health (in Catalan:Pla Estratègic de Recerca i Innovació en Salut)
ProQOL: Professional Quality of Life
